# Drivers of treatment change in PLHIV- psycho-social factors are more important than considerations of adherence

**DOI:** 10.1186/1742-4690-9-S1-P65

**Published:** 2012-05-25

**Authors:** Marian Pitts, Jeffrey Grierson, Rachel Koelmeyer

**Affiliations:** 1 la Trobe University, Melbourne, Australia

## Introduction

There are increasing options available for the effective management of HIV. Treatment regimens have become simpler and more manageable since the introduction of HAART fifteen years ago. In this context it is important to understand how commonly PLHIV switch treatment s and how the decision to change treatment is negotiated between clinician and patient.

## Materials and methods

We conducted an online survey of 254 people living with HIV (PLHIV) in Australia. PLHIV had a median age of 47.5 years. Overall, 87.4% of respondents were currently taking ARV; 5.5% had taken ARV in the past but not currently, and 7.1% had never taken ARV.

## Results

Two-thirds of respondents had changed ARV at some point: 14% in the last 12 months. Respondents last changed treatment a median of 3 years ago. Most respondents (90%) had not used more than six combinations in their lifetime.

Of those who had changed treatment, the most significant driver of the decision was the advice of the physician (76%) the second most important was the side effects of the previous regimen (49%). Fatigue/loss of energy was the most prevalent side effect experienced by 74% of all respondents. It was also rated as the most bothersome on the ACTG-HIS scale.

Aspects associated with adherence were the lowest rated drivers of treatment change. Only 7% of respondents identified problems with taking doses at the correct time, and less than 5% reported missing doses.

Given that the doctor’s advice was the most important driver of treatment change we elicited the important characteristics of the clinician- patient relationship using free text. The doctors’ interpersonal skills and personality was most frequently identified (by 62% of respondents). This was more than double the next most frequently mentioned characteristic (competence- 37%). Open ended contributions from participants also highlighted the importance of open communication and a robust relationship with the clinician.

## Conclusions

A focus on adherence and neglect of relational, psycho-social and somatic factors in assessing motivations for treatment switching is likely to compromise robust patient-clinician relationships.

